# Household air pollution, ultrasound measurement, fetal biometric parameters and intrauterine growth restriction

**DOI:** 10.1186/s12940-021-00756-5

**Published:** 2021-06-23

**Authors:** Anindita Dutta, Donee Alexander, Theodore Karrison, Oludare Morhasson-Bello, Nathaniel Wilson, Omolola Mojisola Atalabi, Damilola Adu, Tope Ibigbami, Samuel Adekunle, Dayo Adepoju, John Olamijulo, Omolola Akinwunmi, Oluniyi S. Afolabi, Oluwafunmilade Deji-Abiodun, Babatunde Adedokun, Briseis Aschebrook-Kilfoy, Oladosu Ojengbede, Christopher O. Olopade

**Affiliations:** 1 Department of Medicine and Center for Global Health, University of Chicago, Chicago, USA; 2 Department of Public Health Sciences, University of Chicago, Chicago, USA; 3 Department of Obstetrics and Gynecology, University of Ibadan, Ibadan, Nigeria; 4 Pritzker School of Medicine, University of Chicago, Chicago, USA; 5 Department of Radiology, University of Ibadan, Ibadan, Nigeria; 6 Healthy Life for All Foundation, House 38, University College Hospital, Ibadan, Nigeria

**Keywords:** Household air pollution, Fetal growth, Ultrasound measurement, Intrauterine growth restriction, Pregnant women, Nigeria

## Abstract

**Background:**

Low birthweight, intrauterine growth restriction (IUGR) and perinatal mortality have been associated with air pollution. However, intervention studies that use ultrasound measurements to assess the effects of household air pollution (HAP) on fetal biometric parameters (FBP) are rare. We investigated the effect of a cookstove intervention on FBP and IUGR in a randomized controlled trial (RCT) cohort of HAP-exposed pregnant Nigerian women.

**Methods:**

We recruited 324 women early in the second trimester of pregnancy. Between 16 and 18 weeks, we randomized them to either continue cooking with firewood/kerosene (control group) or receive a CleanCook stove and ethanol fuel (intervention group). We measured fetal biparietal diameter (BPD), head circumference (HC), femur length (FL), abdominal circumference (AC) and ultrasound-estimated fetal weight (U-EFW) in the second and third trimesters. The women were clinically followed up at six regular time points during their pregnancies. Once during the women’s second trimester and once during the third, we made 72-h continuous measurements of their personal exposures to particulate matter having aerodynamic diameter < 2.5 μm (PM_2.5_). We adopted a modified intent-to-treat approach for the analysis. Differences between the intervention and control groups on impact of HAP on fetal growth trajectories were analyzed using mixed effects regression models.

**Results:**

There were no significant differences in fetal growth trajectories between the intervention and control groups.

**Conclusions:**

Larger studies in a setting of low ambient air pollution are required to further investigate the effect of transitioning to a cleaner fuel such as ethanol on intrauterine growth.

**Trial registration:**

ClinicalTrials.gov NCT02394574; September 2012

## Background

Early-life exposure to environmental contaminants, such as those in household air pollution (HAP), can lead to development of early childhood diseases like asthma, wheezing, respiratory infections, and altered immune defense; also, chronic diseases like diabetes and heart disease [1]. Further, maternal exposure to high levels of air pollutants during pregnancy has been associated with increased risk of adverse pregnancy outcomes such as preterm birth, stillbirth, small-for-gestational-age babies, low birth weight and neonatal death [2–5]. A major source of prenatal and early-life HAP exposure, globally, is the highly prevalent practice of using biomass, kerosene and coal for household cooking and/or heating purposes. Hence, it is necessary to assess fetal health and ultimate birth outcomes among pregnant women exposed to HAP from using biomass, kerosene and coal for cooking. An informative assessment of fetal health is continuous monitoring of fetal growth for the duration of entire pregnancies.

Ultrasound examinations result in reliable and reproducible fetal biometric measurements [6]. Ultrasound is the gold standard approach for measuring fetal growth during pregnancy [7]. The method has been used to examine the impacts of early-life exposure to air pollution on fetal growth. Most such studies have concerned the impact of exposure to ambient air pollution [2, 8–11]. To date, no studies have used ultrasound serially to measure fetal biometric parameters in cook stove intervention trials.

Patelarou and Kelly [12] suggested that fetal growth should be assessed serially during pregnancy rather than only at birth and, further, that direct methods of assessment such as ultrasound provide better insight into the specific effects of maternal exposure to HAP. Therefore, we undertook this study to assess the impact of exposure to HAP in a randomized controlled trial (RCT) of a cook stove fuel intervention, using ultrasound to measure fetal growth during pregnancy. The trial took place in Ibadan, Nigeria, and investigated the impact, on pregnancy outcomes, of transitioning women from cooking with firewood or kerosene to cooking with ethanol. We performed ultrasound measurement of fetal growth six times during the pregnancy on each woman who participated in this study. We hypothesized that the fetuses of the control group women, who were exposed to higher levels of PM_2.5_ from use of firewood and kerosene, would exhibit statistically significant intrauterine growth restriction (IUGR), compared with the fetuses of the intervention group women, who used clean-burning ethanol fuel and, thus, were exposed to lower levels of PM_2.5_.

## Methods

### Study design, participant recruitment and eligibility criteria

In earlier publications we have detailed the processes of participant recruitment and randomization and the eligibility criteria for this RCT [13–15], which was conducted in Ibadan, Nigeria between June 2013 and October 2015. Participants were randomized using randomization module in REDCap to ethanol or control cook stove group. Among 324 participants, half were randomly assigned to control arm (firewood/kerosene), who continued to cook with the fuel they were using at entry, and the other half to ethanol arm, who received CleanCook ethanol stove (CLEANCOOK Sweden AB). Women (*n* = 324), who were apparently healthy, non-smokers and non-chewers of tobacco, cooked regularly with firewood/kerosene, and were at < 18 weeks gestational age, were enrolled to this study. Exclusion criteria included smoking, living with smokers, cooking for a living, HIV-positive status, high-risk pregnancy (multiple gestations, uncontrolled maternal hypertension, maternal age > 35 years for first delivery, three/more prior miscarriages, or a prior Cesarean-section).

With potential recruits, we used a portable ultrasound device to determine gestational age (GA), assembling a sample of 324 participants. Between 16 and 18 weeks fetal GA, we randomized participants either to receive a CleanCook stove and a supply of ethanol fuel (intervention arm, E) throughout pregnancy or to continue cooking with firewood or kerosene (control arm, C). We then followed all participants to term. The average duration of study participation was 156 days.

The study protocol was explained to the participants in detail and their written informed consents were obtained at the time of recruitment. The work has been carried out in accordance with the Code of Ethics of the World Medical Association (Declaration of Helsinki) and the Institutional Review Boards of the University of Chicago and the University of Ibadan approved the study protocol, which was registered as NCT02394574 on ClinicalTrials.gov.

### Questionnaire survey

We gathered information on the participants’ age, level of education, habits, family, dietary details, occupation of the participants, occupation of the spouse and exposure to environmental tobacco smoke (ETS), average family income, cooking hours per day, cooking-years, types of fuel used for cooking, home kitchen design (especially the presence or lack of windows), obstetrics history, current health status, pertinent past medical history and family history, through face-to-face interviews using structured questionnaires in the local language (Yoruba).

### Ultrasound measurements of fetal biometric parameters

After the participating women completed the structured questionnaires and had blood drawn for routine prenatal and malaria screening, they were asked to return to the clinic the following day for the first in a series of ultrasound examinations. All women in the study registered with a clinic midwife who recorded the first day of the women’s last known menstrual period (LMP), expected due date (EDD), and symphysis-fundal height (SFH). A trained radiologist from the University College Hospital (UCH) in Ibadan, Nigeria, who was unaware of the women’s randomization group, performed the appropriate examination to determine estimated GA and other growth parameters in all participants, using a portable Sonosite Micromaxx ultrasound system (Bothell, WA, USA).

### Data collection

We measured and recorded fetal heart rate (FHR), biparietal diameter (BPD), occipitofrontal diameter (OFD), head circumference (HC), abdominal circumference (AC), femur length (FL), amniotic fluid index (AFI), and ultrasound-estimated fetal weight (U-EFW). Additionally, the presence of IUGR, gross fetal anomalies, uterine fibroids, gross uterine or ovarian anomalies, and placental anomalies was recorded. When possible, all measurements were taken three times at each time point and averaged. Fetal weight was estimated using the Hadlock [16] method. In addition to the day after initial clinic presentation, ultrasounds were performed at 20, 26, 30, 34 and 38-weeks GA. The variables analyzed in this report are BPD, HC, FL, AC and U-EFW.

### Personal exposure monitoring

As reported in our prior publications [13–15, 17], we determined duration of cooking using a stove use temperature sensitive monitor, measured pregnant Nigerian women’s levels of personal exposure to PM_2.5_ using RTI MicroPEM for three consecutive days (72 h) at two time points: during the second and third trimesters of pregnancy. Each woman in the study carried the MicroPEM in a small, culturally appropriate bag placed near the breathing zone. An internal accelerometer, which is sensitive enough to detect breathing movement even if the woman was sitting still, served as a quality control check to ensure the monitor was worn. In our analyses, we used the mean PM_2.5_ across both measurement intervals. The summary parameters calculated were mean PM_2.5_, minimum and maximum levels of PM_2.5_ over 72 h, and the time in minutes spent by the women with PM_2.5_ levels above 100 μg/m^3^. We performed PAH in a small subset of the participants and while we observed higher levels in the control group, particularly kerosene users, the numbers were too small to make any definitive and conclusive comments based on the measurements.

### Data analysis

**Fig. 1:**
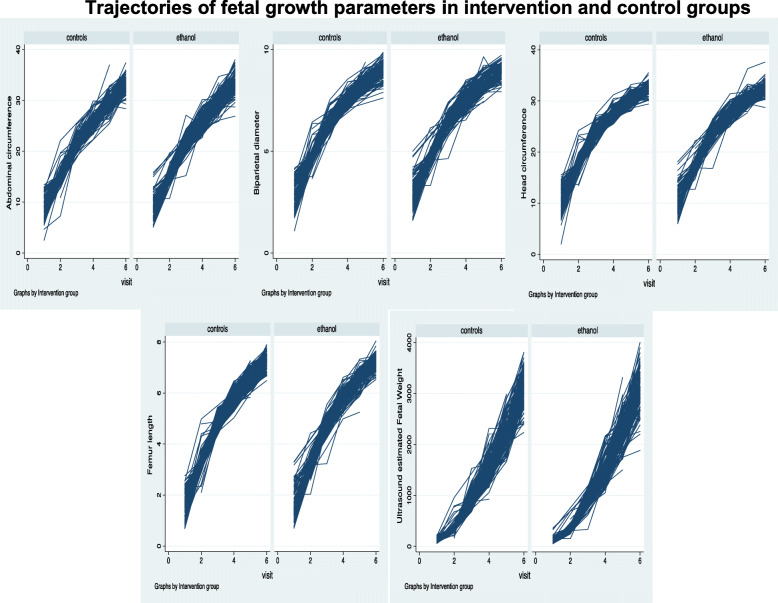
Trajectories of fetal growth parameters in intervention and control groups

Our analysis employed the modified intent-to-treat (ITT) approach, as the final analysis excluded some participants that had no follow up data after randomization into the study groups. However, for the analysis, participants were included in the group to which they were randomized regardless of stove use compliance according to the intention to treat principle. The longitudinal fetal outcome variables analyzed were BPD, HC, FL, AC and U-EFW at time of recruitment and at each of the five subsequent time points across pregnancy: GA 20, 26, 30, 34 and 38 weeks. Analysis focused on the difference in growth patterns between the intervention (ethanol) and control (firewood/kerosene) groups. We used mixed effects models for analysis of intrauterine growth variables. For each of the growth parameters, we fitted a random coefficient model regressing the repeat measurements on intervention group in one set of analyses and on PM_2.5_ measurements in another. The random coefficient model treated subjects as a random effect allowing for correlation between repeat measurements of fetal growth. Additionally, the trajectories of fetal parameters (Fig. 1) indicated a non-linear trajectory as growth rate was initially high and then slowed (although the reverse was found for U-EFW), thus, a quadratic term was included for GA. The observation that some fetuses were consistently bigger than others suggested a random intercept model was indicated, while a random slope was added to the model to also allow for fetuses to differ in their overall rate of growth concerning intrauterine growth parameters.

Letting y_ij_ denote fetal intrauterine growth measurements for subject j at visit i, the random intercept model was:

Y_ij_ = β_1_ + β_2_x_ij_ + β_3_x_ij_^2^ + β_4_Z_j_ + ζ_1j_ + ζ_2j_x_ij_ + ε_ij_ [1].

x_ij_ is the GA for subject j at visit i.

Z_j_ is a dummy variable for the intervention.

ζ_1j_ is the random intercept.

ζ_2j_ is the random slope of GA.

ε_ij_ is the GA specific error term that allows the growth measurements to deviate from the perfectly quadratic trajectories defined by the first four terms.

We assessed the potential importance of adding the quadratic term by examining the estimates and associated *p*-values of the square of the GA variable included in the model. In a second model, the dummy variable for the intervention was replaced with a four-category variable (quartiles) for measured PM_2.5_. The quartiles were determined based on the distribution of PM_2.5_ among controls. The random coefficient models were adjusted for mother’s age, body mass index, marital status, educational level, number of children, history of stillbirth, and history of miscarriage. Regression coefficients and 95% confidence intervals were reported.

## Results

The baseline clinical and demographic characteristics were similar between the study groups and have been reported in our previous publications [13, 14]. Overall, few women were diabetic (1.2% ethanol-users and 1.9% firewood/kerosene users). The mean age of the mothers was 28 years (range 14–44) and mean body mass index was 24 kg/m^2^ (range 14.2–45.0). Education levels varied from none to beyond high school. Many participants were illiterate in both groups (37.9% ethanol users and 43.2% firewood/kerosene users). Mean GA at entry was 13 weeks, ranging from 6.7–18 weeks. We did not randomize any participant to the study’s intervention or control arm until 16 weeks GA. The majority of the women had two or fewer children at enrollment. Over 25% of them had a prior miscarriage and 8% a prior stillbirth. Of the 324 enrolled participants, 306 completed the study, while 18 participants dropped out between randomization and delivery (8 intervention; 10 control). Table 1 shows the summary data for exposure levels for the intervention (ethanol) and control (firewood/kerosene) groups.

**Table 1:** Summary statistics for PM_2.5_ levels in the study groups and overall

	Intervention		Control		All	
2nd trimester mean 72-h PM_2.5_ quartile	Median (min, max)	n	Median (min, max)	n	Median (min, max)	n
Q1	20.3 (5.5–25.1)	32	21.5 (12.8–26.6)	26	20.6 (5.5–26.6)	58
Q2	32.3 (27–40.0)	29	33.0 (27.0–40.5)	29	32.9 (27.0–40.5)	58
Q3	52.5 (40.9–66.5)	28	51.4 (41.3–67.6)	30	51.8 (40.9–67.6)	58
Q4	166.3 (69.6–437.8)	27	161.5 (69.6–437.8)	31	162.6 (69.6–437.8)	58
3rd trimester mean 72-h PM_2.5_ quartile						
Q1	26.1 (0.7–32.3)	28	22.0 (8.8–31.3)	24	23.8 (0.7–32.3)	52
Q2	39.7 (32.7–48.2)	26	40.9 (33.9–48.9)	26	40.1 (32.7–48.9)	52
Q3	62.8 (49.2–106.8)	27	71.5 (49.5–113.8)	25	64.5 (49.2–113.8)	52
Q4	189.8 (122.2–890.5)	24	186.3 (117.1–698.6)	27	186.4 (117.0–890.5)	51

*n* sample size; *min* minimum; *max* maximum

**Fig. 2:**
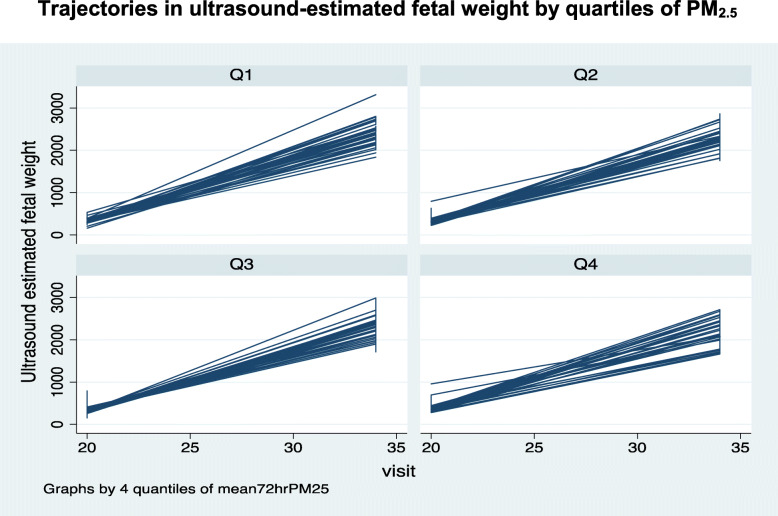
Trajectories in ultrasound-estimated fetal weight by quartiles of PM_2.5_

The changes in the fetal parameters by group (intervention/control) are shown in Table 2 and Fig. 1. The growth trajectories appear similar between the two groups and there were no significant differences between the intervention and the control groups in the random effects models (Table 3). However, a borderline significant difference in growth parameter by quartiles of PM_2.5_ was found in the random effects model for U-EFW. Those in the fourth quartile of mean PM_2.5_ had a significantly higher mean U-EFW compared to those in the first quartile (*p* = 0.046) (Table 4, Fig. 2).

**Table 2:** Summary statistics for fetal parameters by gestational age and group (control/intervention)

			Control					Intervention		
**Gestational age (weeks)**	**BPD**	**HC**	**FL**	**AC**	**U-EFW**	**BPD**	**HC**	**FL**	**AC**	**U-EFW**
13 weeks										
Mean	2.9	10.7	1.6	8.9	120.9	3.0	11.0	1.7	9.1	125.4
SD	0.7	2.6	0.6	2.3	48.5	0.7	2.5	0.6	2.3	60.2
n	108	103	103	104	97	104	100	99	100	95
20 weeks										
Mean	4.8	17.6	3.3	15.2	357.9	4.7	17.4	3.3	15.1	340.4
SD	0.4	1.3	0.3	1.4	97.6	0.3	1.0	0.3	1.1	57.4
n	154	154	154	154	150	147	147	147	147	147
26 weeks										
Mean	6.5	24.0	4.9	21.6	911.2	6.5	23.8	4.8	21.5	887.3
SD	0.3	1.1	0.2	1.2	135.4	0.3	1.1	0.3	1.3	120.0
n	145	145	145	145	143	148	148	148	148	145
30 weeks										
Mean	7.6	27.7	5.8	25.9	1525.0	7.5	27.7	5.8	25.9	1529.2
SD	0.3	0.9	0.3	1.2	203.5	0.4	1.2	0.3	1.5	223.5
n	145	145	145	145	144	149	149	149	149	148
34 weeks										
Mean	8.4	30.5	6.6	29.8	2295.6	8.4	30.5	6.6	29.9	2315.1
SD	0.3	1.0	0.3	1.6	277.0	0.4	1.2	0.3	1.5	288.2
n	140	140	139	140	140	150	150	150	150	148
38 weeks										
Mean	8.9	32.4	7.3	33.1	3061.7	8.9	32.4	7.2	33.1	3042.7
SD	0.4	1.1	0.3	1.6	324.3	0.4	1.2	0.3	1.9	374.2
n	113	113	113	113	112	120	120	120	120	116

*n* number; *SD* standard deviation; *BPD* biparietal diameter; *HC* head circumference; *FL* fetal length; *AC* abdominal circumference; *U-EFW* ultrasound-estimated fetal weight

**Table 3:** Regression of fetal parameters on group random intercept model*

Fetal parameters	Coefficient	95% Confidence Interval	***p***-value
BPD			
Randomization group	− 0.010	− 0.066 to 0.046	0.736
Gestational age (weeks)	0.390	0.375 to 0.405	< 0.001
Gestational age^2^	− 0.003	− 0.003 to − 0.002	< 0.001
HC			
Randomization group	0.008	−0.185 to 0.169	0.926
Gestational age (weeks)	1.504	1.450 to 1.557	< 0.001
Gestational age^2^	−0.012	−0.013 to − 0.011	< 0.001
FL			
Randomization group	−0.013	−0.060 to 0.034	0.586
Gestational age (weeks)	0.313	0.301 to 0.325	< 0.001
Gestational age^2^	−0.002	−0.002 to − 0.001	< 0.001
AC			
Randomization group	0.027	−0.199 to 0.253	0.817
Gestational age (weeks)	0.988	0.933 to 1.044	< 0.001
Gestational age^2^	0.0002	−0.001 to 0.001	0.747
U-EFW			
Randomization group	−5.336	−27.589 to 16.917	0.638
Gestational age (weeks)	−120.744	− 127.529 to −113.959	< 0.001
Gestational age^2^	4.738	4.610 to 4.866	< 0.001

*SE* standard error; *BPD* biparietal diameter; *HC* head circumference; *FL* fetal length; *AC* abdominal circumference; *U-EFW* ultrasound-estimated fetal weight

*Adjusted for mother’s age, body mass index, marital status, educational level, number of children, history of stillbirth, and history of miscarriage

**Table 4:** Regression of fetal parameters on PM_2.5_ exposure levels*

Fetal parameters	Coefficient	95% Confidence Interval	***p***-value
BPD			
Mean PM_2.5_ quartile*			
Q2	−0.001	−0.098 to 0.096	0.985
Q3	0.040	−0.058 to 0.138	0.424
Q4	0.036	−0.063 to 0.135	0.474
HC			
Mean PM_2.5_ quartile			
Q2	−0.065	− 0.394 to 0.264	0.698
Q3	0.021	−0.311 to 0.353	0.901
Q4	0.102	−0.232 to 0.435	0.551
FL			
Mean PM_2.5_ quartile			
Q2	−0.057	− 0.140 to 0.024	0.168
Q3	−0.012	−0.093 to 0.070	0.780
Q4	0.007	−0.075 to 0.089	0.869
AC			
Mean PM_2.5_ quartile			
Q2	−0.216	− 0.583 to 0.151	0.248
Q3	0.063	−0.306 to 0.432	0.738
Q4	0.083	−0.291 to 0.457	0.663
U-EFW			
Mean PM_2.5_ quartile			
Q2	−1.02	−31.144 to 29.112	0.947
Q3	14.13	−16.749 to 45.015	0.370
Q4	**32.59**	**0.559 to 64.628**	**0.046**

*BPD* biparietal diameter; *HC* head circumference; *FL* fetal length; *AC* abdominal circumference; *U-EFW* ultrasound-estimated fetal weight; Reference category for PM_2.5_ level is the first quartile; square of gestational age was omitted from the model; *reference category – first quartile

*Adjusted for mother’s age, body mass index, marital status, educational level, number of children, history of stillbirth, and history of miscarriage

## Discussion

To the best of our knowledge, this study is the first randomized controlled, ethanol fuel cookstove intervention conducted with the intent of characterizing the effect of HAP exposure reduction on birth outcomes and monitoring personal exposures to PM_2.5_ during pregnancy in the context of an ethanol cook stove intervention. Additionally, it is the first to have used ultrasonography to make repeated (six) periodic measurements of fetal biometric parameters, including BPD, HC, FL, AC and U-EFW in a HAP intervention study cohort. No significant differences in growth trajectories between the intervention and control groups were observed in the random effects models. Additionally, there were no significant associations between PM_2.5_ levels and fetal parameters, although a borderline significantly higher U-EFW at the highest quartile was in the unexpected direction, suggesting this could have been a spurious finding. We note that the study area—Ibadan, Nigeria—has relatively high ambient air pollution levels and infer that this may have played a role in minimizing any effects of the ethanol stove intervention. However, we advocate that serial ultrasound monitoring of FBP may help to identify IUGR earlier during pregnancy for women exposed to HAP.

Exposure to HAP derived from the use of biomass and kerosene for cooking has been linked to many health problems. Women and children in low-income countries disproportionately suffer from the consequences of HAP exposure, as they are most often tasked with meal preparation. In urban and peri-urban areas of Nigeria where this study was conducted, households predominantly utilize biomass (wood) and kerosene for cooking, with the proportion using kerosene as high as 55%. This is because cleaner burning fuels such as propane are expensive and so not affordable.

Many studies have suggested a link between HAP exposure in pregnant women and the frequency of stillbirth, pre-term delivery, and low-birth weight [2–4, 18]. These studies showed that mean birth weight is lower in households utilizing biomass fuels for cooking and even lower in households utilizing kerosene. However, most of these studies were cross-sectional in design and relied on survey instruments such as census or community health surveys. A few measured pollution levels in the kitchen but did not perform personal exposure monitoring in the pregnant women. Further, these studies are plagued by the residual confounding inherent in any cross-sectional design.

While associations between HAP exposure and adverse pregnancy outcomes have been reported, there has been little serial monitoring of IUGR using ultrasound. Thus, there is a paucity of data relating fetal growth patterns to in-utero personal exposure to HAP. Our study, which transitioned pregnant women from using kerosene and firewood to a cleaner fuel, ethanol, fills this gap by demonstrating that HAP exposure from cooking with dirty fuels is associated with adverse pregnancy outcomes [14], though the intervention did not appear to have an effect on intrauterine fetal growth.

Although our study did not find a significant effect of the intervention on fetal growth, we recommend that for future randomized controlled studies that involve an intervention to reduce pregnant women’s exposures to HAP, specifically, it may be necessary to select locations where population density is lower, ambient air pollution levels are lower, and homes are separated by larger distances. This would reduce the potential for ambient air pollution and HAP from nearby houses to influence exposure level values observed from personal monitoring of individual women in their homes.

Performing ultrasound measurements at the time of recruitment and repeating these measures at GA 20, 26, 30, 34 and 38-weeks during the entire pregnancy, personal monitoring of HAP exposure twice during pregnancy, and using robust statistical measures are the strengths of this study. However, there are some limitations. First, in Nigeria, women generally don’t present for antenatal care until late in the second trimester. For this reason, we were unable to determine fetal growth rate during the first trimester. Fetal growth was not determined until participants were randomized during their second trimesters, between 16 and 18 weeks GA. Second, as mentioned earlier, relatively high background ambient air pollution levels in Ibadan may have minimized any beneficial effects of the ethanol stove intervention on fetal development.

Despite the above-mentioned limitations, this study stands out as the first one to utilize a sensitive tool like ultrasonography to measure the early effects of HAP exposure reduction on fetal biometric measures in a cohort of Nigerian women. Our study makes a significant contribution to the literature on the health implications of HAP exposure for pregnant women.

Assessing in-utero growth deficits due to exposure to environmental pollutants at different time points during pregnancy is of great importance from the scientific and public health perspectives. Substantial reduction in in-utero exposure to HAP, to levels below the standard limits set by the World Health Organization (WHO) is important in order to promote healthy fetal growth. However, our ethanol fuel intervention was not significantly associated with better fetal growth outcomes than were observed in the control group (kerosene/firewood fuel). Larger studies with Tier 4 cooking stoves/fuels are required to augment our findings related to the health effects of HAP exposure reduction. Robust results obtained from larger studies could drive policy and business decisions in countries dealing with the adverse effects of air pollution.

## Conclusions

Our study demonstrated that fetal growth parameters were not significantly different between women randomized to an ethanol cookstove intervention and those that continued to use kerosene or firewood.

## Data Availability

The datasets generated and analyzed during the current study are not publicly available because they are small data generated from the cohort but are available from the corresponding author on reasonable request.

## Abbreviations

GA: Gestational age
HAP: Household air pollution
RCT: Randomized controlled trial
E: Ethanol
C: Control
FHR: Fetal heart rate
BPD: Biparietal diameter
OFD: Occipitofrontal diameter
HC: Head circumference
AC: Abdominal circumference
FL: Femur length
AFI: Amniotic fluid index
U-EFW: Ultrasound-estimated fetal weight
LMP: Last known menstrual period
EDD: Expected due date
SFH: Symphysis-fundal height
UCH: University college hospital
ITT: Intent-to-treat
HPCS: Haifa pregnancy cohort study
WHO: World health organization
